# Effects of licorice extract in combination with a low-calorie diet on obesity indices, glycemic indices, and lipid profiles in overweight/obese women with polycystic ovary syndrome (PCOS): a randomized, double-blind, placebo-controlled trial

**DOI:** 10.1186/s13048-024-01446-9

**Published:** 2024-07-30

**Authors:** Hadis Hooshmandi, Akram Ghadiri-Anari, Ali Mohammad Ranjbar, Hossein Fallahzadeh, Mahdieh Hosseinzadeh, Azadeh Nadjarzadeh

**Affiliations:** 1 Research Center for Food Hygiene and Safety, School of Public Health, Shahid Sadoughi University of Medical Sciences, Yazd, Iran; 2 Department of Nutrition, School of Public Health, Shahid Sadoughi University of Medical Sciences, Yazd, Iran; 3 Diabetes Research Center, Shahid Sadoughi University of Medical Sciences, Yazd, Iran; 4 Department of Pharmacognosy, Faculty of Pharmacy, Shahid Sadoughi University of Medical Sciences, Yazd, Iran; 5 Herbal Medicine Center, Faculty of Pharmacy, Shahid Sadoughi University of Medical Sciences, Yazd, Iran; 6 Department of Biostatistics and Epidemiology, Research Center of Prevention and Epidemiology of Non-Communicable Disease, School of Public Health, Shahid Sadoughi University of Medical Sciences, Yazd, Iran

**Keywords:** Polycystic ovary syndrome, Licorice, Insulin, FBS, Lipid, LDL, HDL, TG

## Abstract

**Background:**

Polycystic ovary syndrome (PCOS) is the most common ovarian dysfunction. Recent studies showed the effectiveness of licorice on metabolic profiles with inconsistent findings. So, we investigated the effect of licorice on obesity indices, glycemic indices, and lipid profiles in women with PCOS.

**Methods:**

This randomized, double-blind, placebo-controlled trial was performed on 66 overweight/obese women with PCOS. The participants were randomly assigned to receive either 1.5 gr/day licorice extract plus a low-calorie diet (*n* = 33) or placebo plus a low-calorie diet (*n* = 33) for 8 weeks. Participants’ anthropometric indices and body composition were assessed using standard protocols. Fasting blood sugar (FBS), insulin levels, low-density lipoprotein-cholesterol (LDL-C), total cholesterol (TC), triglyceride (TG), and high-density lipoprotein-cholesterol (HDL-C) were measured using enzymatic kits. The homeostasis model assessment-insulin resistance (HOMA-IR) and HOMA of β-cell function (HOMA-B) were calculated using valid formulas.

**Results:**

Between-group comparisons demonstrated significant differences between the groups in terms of obesity indices (body weight, BMI, and body fat), lipid profiles (TG, TC, LDL-C, and HDL-C), FBS and insulin levels, HOMA-IR, and HOMA-B at the end of the study (*P* < 0.05). Supplementation with licorice plus a low-calorie diet was also more effective in improving all parameters than a low-calorie diet alone after adjusting for confounders (baseline values, age, weight changes, and physical activity changes) (*P* < 0.05).

**Conclusion:**

The findings showed that licorice consumption leads to improvements in obesity indices, glucose homeostasis, and lipid profiles compared to placebo. Due to possible limitations of the study, further research is needed to confirm these findings.

## Introduction

Polycystic ovary syndrome (PCOS) is a metabolic and reproductive disorder affecting 6–12% of women of reproductive age [1]. Previous research showed that 13.6%, 19.4%, and 17.8% of Iranian women have PCOS according to the diagnostic criteria of the National Institutes of Health, Rotterdam, and Androgen Excess Society, respectively [2]. The main clinical manifestations of PCOS are oligo-amenorrhea or amenorrhea, hyperandrogenism or hyperandrogenemia, and sonographic findings of polycystic ovaries [3]. PCOS can present with various signs and symptoms, but its most prevalent complications include acne, hirsutism, obesity, type 2 diabetes, insulin resistance (IR), glucose intolerance, and dyslipidemia [4]. Obesity is strongly associated with PCOS as between 30 and 75% of people with PCOS are obese [5]. Obese women with PCOS have higher IR than healthy women with the same body mass index (BMI) [6]. There is evidence that hyperinsulinemia, which is associated with compensatory IR and elevated blood glucose, plays a major role in this disease [7]. IR increases the levels of androgen hormones in the body and makes weight loss difficult in these patients [8].

Lifestyle-based interventions such as dietary recommendations that induce weight loss and increase insulin sensitivity might be helpful in the management of the disease [9, 10]. Obese and overweight subjects often turn to drugs and supplements to maintain weight due to difficulty adhering to dietary recommendations. Recently, medicinal plants have become more widely used for weight loss [11]. A common herbal medicine is licorice, G. glabra root, which has a variety of therapeutic and medicinal properties, including antiviral, anti-peptic ulcer, antioxidant, and anti-inflammatory properties [12]. The beneficial effects of licorice are attributed to its major ingredients, such as glycyrrhizinic acid, glabridin, and iso-liquiritigenin [12]. Interestingly, patients suffering from chronic diseases have been shown to benefit from licorice and its components. In this case, a systematic review and meta-analysis showed that licorice supplementation significantly reduced body weight and BMI [13]. However, inconsistent results were found regarding the effects of supplementing with licorice on lipid profiles [14–17]. Accordingly, the beneficial effects of licorice supplementation on total cholesterol (TC) and low-density lipoprotein cholesterol have been shown in some studies [14–16], while others did not find any effects [17]. Furthermore, two clinical studies showed that licorice [18] and glabridin, a major component of licorice [19], supplementation significantly improved IR and fasting insulin concentrations. However, it should be noted that in a study by Namazi et al., while licorice supplementation improved IR in the intervention group, the effect wasn’t significant compared to the placebo (low-calorie diet) group [18].

Despite the importance of metabolic changes in the development of PCOS, there are few human studies on the effect of licorice or its components on the metabolic profiles in this syndrome [19]. Also, as far as we know no study investigated the effects of licorice supplementation in combination with a low-calorie diet on metabolic profiles in women with PCOS. Therefore, in this study, we investigated the effects of licorice and weight loss diet on obesity indices, glycemic and lipid profiles in overweight/obese women with PCOS.

## Materials and methods

### Study design

This 8-week randomized, double-blind, placebo-controlled trial involved 72 overweight/obese women with PCOS (aged 18–45 years old) referred to Emam Ali outpatient clinic, Yazd, Iran. (IRCT20200922048802N2). Samples were selected by convenience sampling method and eligible patients were divided into two groups using a random number table. Accordingly, the intervention group received three capsules of licorice extract (*n* = 36) and the placebo group received three capsules of placebo (contained corn starch) (*n* = 36). Both groups also followed a low-calorie diet during the study period.

### Population (inclusion and exclusion criteria)

Inclusion criteria for this study were age 18–45 years, BMI 25–35 kg/m2, and having PCOS based on the Rotterdam criteria. The participants were diagnosed to have PCOS if they had at least two of the following three criteria: (A) no ovulation or oligo-ovulation (or the number of monthly cycles less than 6 cycles during 12 months), (B) clinical or biochemical signs of androgen elevations: including acne, hirsutism, alopecia, or elevated serum androgen levels, and (C) polycystic ovary with 12 or more follicles in each ovary with a diameter between 2 and 3 mm or increasing ovarian volume more than 11 cubic centimeters. Subjects were not included in the study if they had blood pressure greater than or equal to 140/90 mmHg, had cardiovascular, liver, kidney, diabetes, and thyroid disease with regular drug use, were smoking, took vitamin-mineral, antioxidant, and herbal supplements during the previous 3 months, consumed licorice more than 300 g per week, were pregnant or lactating women, and adhered to a special diet or exercise. They also were not included if they had other causes of hyperandrogenism (hereditary adrenal hyperplasia, Cushing’s syndrome, hyperprolactinemia, and hypothyroidism), androgen-secreting tumors, and a history of allergy to licorice or any of its components. In terms of medication, patients who had previously received metformin continued to take it, but patients taking estrogen and progesterone were not included in the study. Moreover, patients who were treated with new drugs from two months before and during the study were not included in this study. The study protocol was confirmed by the Ethics Committee of Shahid Sadoughi University of Medical Sciences (IR.SSU.SPH.REC.1397.152) and the participants filled in an informed consent form before participating in the study.

### Intervention groups and execution of the study

In this study, the intervention group received three capsules of licorice extract supplement following a low-calorie diet (*n* = 36). To encapsulate licorice extract, spray dried (Sprayed) licorice extract powder was purchased from Shirin Daroo Company, Shiraz, Iran, and after confirming its analysis by a pharmacognosy specialist, the packaging was done in the Faculty of Pharmacy, Shahid Sadoughi University of Medical Sciences, Yazd, Iran. Each licorice capsule contained 500 mg of licorice and 7.03% glycyrrhizic acid (36.5 mg). The placebo group also received 3 capsules of corn starch following a low-calorie diet (*n* = 36). Each placebo capsule contained 500 mg of inert substance (corn starch).

As mentioned, study participants consumed three capsules of licorice at a dose of 500 mg per day for 8 weeks, half an hour before each main meal, in which case 168 tablets were allocated to each person. At the beginning of the study, 84 pills, which is a 4-week quota for each person, were given to individuals. After this course, participants were contacted and invited to receive the second quota. During the second session, the number of unused package houses was counted and recorded. At the end of the second session, the next 4 weeks’ quota was given to each person. The third session was held after the study and the number of unused capsules was counted by each person. To prevent falls to dropouts, people were contacted every week and asked about the consumption process. In each session, blood pressure was measured with a digital blood pressure monitor. Participants were asked not to change their physical activity, medication, or lifestyle during the project. If the person had taken less than 70% of the pills, she was excluded from the study. A two-day 24-hour food recall questionnaire was completed by a nutritionist at the beginning and end of the study to estimate energy intake, macronutrients, and micronutrients.

In this study, both groups were given the same low-calorie diet. The characteristic of the diet used in this plan was to reduce energy intake by ~ 500 kcal from the total energy requirement. A person’s energy level also varied and was usually set between 1,200 and 1,800 kcal per day. The carbohydrates in the diet were adjusted to about 50 to 55% of the total energy, and sources such as grains, fruits, vegetables, and legumes were used. The protein of this diet was considered to be 15 to 25% of the total calories. Dietary fat was adjusted to about 30%. Increasing fiber intake was recommended to reduce the individual’s dietary density and delay gastric emptying. Alcohol and foods high in sugar were limited. At each visit, which was performed once every 4 weeks, the rate of adherence to the diet was checked by a 24-hour recall and 2 days of food record.

### Determination of basic data

In this study, after obtaining written consent, the participant’s general information including age, sex, and history of diseases was collected. Before and after the intervention, anthropometric indices including weight, height, waist circumference (WC), and hip circumference (HC) were measured based on the standard protocols. To assess the level of physical activity, the Beck Questionnaire was used by interviewing at the beginning and end of the study [20]. A blood sample was taken from the participants at the beginning and end of the study to measure biochemical parameters.

### Anthropometric and body composition measurements

The body weight of patients with minimal clothing and without shoes was measured with a digital scale with a sensitivity of 100 g. The height of individuals was measured using a tape measure standing on the wall without shoes with an accuracy of 0.1 cm. The BMI was calculated based on the formula of weight in kilograms divided by height squared in meters. The WC was measured transversely with a non-elastic tape as the minimum radius of distance from the umbilicus to the end of the sternum and HC as the maximum diameter in the hip area transversely [21]. Body composition was measured using the InBody 270 body composition analyzer.

### Biochemical evaluation

Following fasting for 8 h, 5 cc of venous blood was collected from the patients and kept at -73 °C after serum separation. Blood samples were collected to determine the levels of insulin hormone, fasting blood glucose (FBG), LDL-C, high-density lipoprotein-cholesterol (HDL-C), triglyceride (TG), and TC, once at the beginning of the study and again at the end. Serum levels of FBS, LDL-C, HDL-C, TG, and TC were measured using an auto-analyzer (Abbot Model Aclyon 300 USA) by a commercial enzymatic kit (Pars Azmoon, Iran). Enzyme-linked immunosorbent assay (ELISA) method was used to determine insulin levels (Pars Azmoon, Iran). The homeostasis model assessment-insulin resistance (HOMA-IR) was evaluated based on FBS and insulin levels as follows: HOMA-IR = fasting glucose (mg/dL) ×fasting insulin (mU/L) /405 [22]. Also, HOMA of β-cell function (HOMA-B) index, as a good measure of β-cell function, was calculated as follows:

HOMA-B = 20 × fasting insulin (µU/ml)/ fasting glucose (mmol/l) – 3.5 [23].

### Statistical analysis

The normal distribution of continuous variables was checked using Kolmogorov-Smirnov test. Independent sample t-test or Mann-Whitney test was used for comparison of continuous variables between the two groups. Analysis of covariance (ANCOVA) was used for comparisons of continuous variables between the two groups after adjusting for baseline values and other confounders such as age, weight changes, and physical activity changes. Paired t-test was used to compare within-group differences from baseline to post-intervention. The data were analyzed using SPSS software (version 24, SPSS Inc., Chicago, IL, USA) and *P* < 0.05 was considered statistically significant.

## Results

**Fig. 1:**
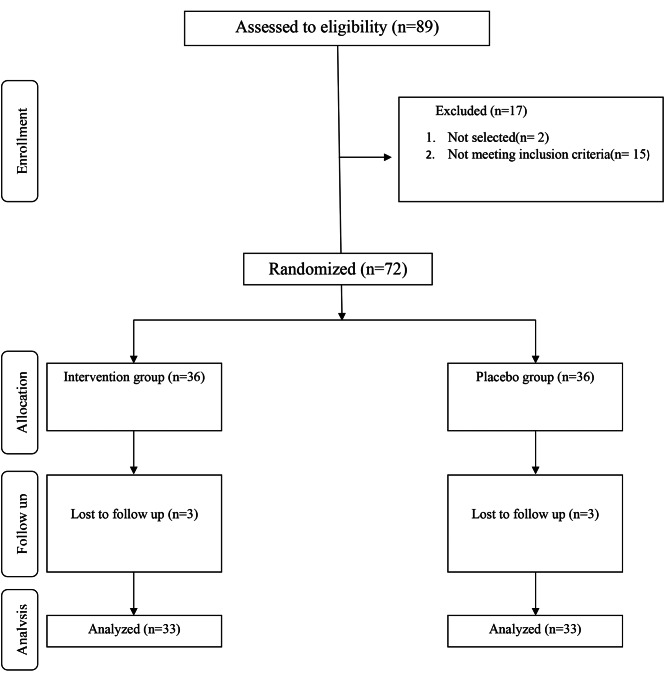
Summary of patient flow diagram

In this study, seventy-two overweight/obese women with PCOS patients were enrolled. They were randomly assigned to either the licorice group (*n* = 36) or the placebo group (*n* = 36). During the study, 6 participants (three in each group) failed to follow the study procedure and were excluded from the final analyses. So, the analyses were performed on 33 participants in each group (Fig. 1**)**. During this study, one patient from the intervention group (one of 36 participants) was excluded from the study due to a change in her diet. Regarding consumption of pills, the adherence rate was 100% and all pills were consumed by the participants completely. If the person had taken less than 70% of the pills, she should have been excluded from the study.

As shown in Table 1, There were no significant differences between the two groups in terms of baseline characteristics. Changes in dietary intakes of the participants during the study are presented in Table 2. As shown, the dietary intakes of the participants were not statistically significant between the groups at baseline and after 8 weeks of intervention (*p*˃0.05). Also, the physical activity habits of the participants were not statistically significant within and between the groups at baseline and after 8 weeks of intervention (P˃0.05, results didn’t show).

**Table 1:** Baseline characteristics of overweight/obese women with PCOS in intervention and placebo groups

Variable	Intervention group (*n* = 33)	Control group (*n* = 33)	*P*-value^*^
**Age (year)**	41.55 ± 9.17	39.97 ± 10.29	0.70
**Weight (kg)**	88.85 ± 4.83	87.93 ± 4.12	0.41
**Height (cm)**	158.71 ± 6.33	157.35 ± 7.95	0.61
**Body mass index (kg/m2)**	32.29 ± 2.75	31.62 ± 2.08	0.23
**Systolic blood pressure (mmHg)**	121.01 ± 10.16	118.92 ± 11.45	0.82
**Diastolic blood pressure (mmHg)**	84.41 ± 5.23	81.19 ± 6.53	0.08
**Heart beat (Beats/min)**	82.32 ± 10.28	79.32 ± 11.67	0.43
**Total physical activity score**	6.61 ± 1.05	6.78 ± 0.78	0.47

− Data are presented as mean ± SD.

− ^*^ Independent sample t-test or Mann-Whitney test were used for comparison of quantitative variables between the two groups.

− *P* < 0.05 was considered statistically significant.

**Table 2:** Dietary intakes of overweight/obese women with PCOS before and after 8 weeks of intervention

Variables	Measurement period	Intervention (*n* = 33)	Control (*n* = 33)	*P*-Value^*^
**Energy (Kcal)**	Before	1904.78 ± 38.40	1901.21 ± 43.38	0.72
	After	1378.18 ± 50.66	1385.09 ± 41.25	0.55
**Carbohydrate (g/d)**	Before	261.27 ± 5.19	260.66 ± 5.88	0.69
	After	188.85 ± 6.94	189.79 ± 5.73	0.46
**Protein (g/d)**	Before	99.54 ± 2.01	99.60 ± 2.27	0.81
	After	72.03 ± 2.65	72.54 ± 2.20	0.45
**Total fat (g/d)**	Before	50.54 ± 1.09	50.33 ± 1.29	0.53
	After	36.48 ± 1.34	36.73 ± 1.10	0.51
**SFA (g/d)**	Before	10.54 ± 0.20	10.55 ± 0.24	0.54
	After	7.66 ± 0.31	7.69 ± 0.23	0.56
**MUFA (g/d)**	Before	8.66 ± 0.17	8.64 ± 0.17	0.71
	After	6.26 ± 0.23	6.30 ± 0.19	0.55
**PUFA (g/d)**	Before	18.14 ± 0.36	18.11 ± 0.41	0.78
	After	13.13 ± 0.48	13.20 ± 0.40	0.55
**Vitamin A (mg)**	Before	464.58 ± 9.36	463.70 ± 10.58	0.73
	After	336.14 ± 12.35	337.80 ± 10.06	0.55
**Vitamin E (mg)**	Before	16.01 ± 0.33	15.98 ± 0.36	0.72
	After	11.39 ± 0.42	11.45 ± 0.34	0.54
**Vitamin D (mg)**	Before	4.24 ± 0.08	4.23 ± 0.11	0.74
	After	3.07 ± 0.12	3.09 ± 0.09	0.55
**Vitamin C (mg)**	Before	66.57 ± 5.65	64.52 ± 4.54	0.71
	After	51.18 ± 2.56	54.53 ± 3.45	0.54
**Fiber (g/d)**	Before	21.65 ± 0.44	21.61 ± 0.49	0.72
	After	15.49 ± 0.57	15.57 ± 0.47	0.55

− The data are presented as means ± SD.

− SFA: Saturated fatty acid; MUFA: Mono unsaturated fatty acid; PUFA: Poly unsaturated fatty acid.

− * Independent sample t-test or Mann-Whitney test were used for comparison of quantitative variables between the two groups.

− *P* < 0.05 was considered statistically significant.

Table 3 shows the effects of licorice supplementation on obesity indices, blood biomarkers, and insulin resistance in overweight/obese women with PCOS. At baseline, there were significant differences between the groups in terms of percent body fat (PBF) (*p*˃0.05), HDL-C (*p* = 0.01), and HOMA-B (*p* = 0.01). After 8 weeks of intervention, the intervention group showed significant improvements in obesity indices (body weight, BMI, and PBF), lipid profiles (TG, TC, LDL-C, and HDL-C), FBS, insulin levels, and HOMA-IR and HOMA-B (*p* < 0.001). The low-calorie diet did not lead to significant changes in the control group after 8 weeks (*p*˃0.05). Between-group comparisons demonstrated significant differences between the groups in terms of obesity indices (body weight, BMI, and PBF), lipid profiles (TG, TC, LDL-C, and HDL-C), FBS and insulin levels, HOMA-IR, and HOMA-B at the end of the study (*p* < 0.05). Supplementation with licorice plus a low-calorie diet was also more effective in improving all parameters than a low-calorie diet alone after adjusting for confounders (baseline values, age, weight changes, and physical activity changes) (*p* < 0.05).

**Table 3:** The effects of licorice supplementation on obesity indices, blood biomarkers, and insulin resistance in overweight/obese women with PCOS

Variables	Intervention group (*n* = 33)	Control group (*n* = 33)	*P-value	^**^P-value	^***^P-value
**Body weight (kg)**					
Baseline	88.84 ± 4.83	87.94 ± 4.12	0.54		
After intervention	82.60 ± 4.30	86.09 ± 10.71	< 0.001	< 0.001	< 0.001
****P-value	< 0.001	0.41			
**Body mass index (kg/m2)**					
Baseline	30.67 ± 0.53	30.72 ± 0.48	0.43		
After intervention	29.03 ± 0.60	30.73 ± 0.47	< 0.001	< 0.001	< 0.001
****P-value	< 0.001	0.25			
**Percent body fat (%)**					
Baseline	30.66 ± 1.47	29.76 ± 0.75	0.01		
After intervention	28.21 ± 0.65	29.75 ± 0.75	< 0.001	< 0.001	< 0.001
****P-value	< 0.001	0.97			
**Triglyceride (mg/dl)**					
Baseline	144.98 ± 39.80	140.00 ± 41.74	0.62		
After intervention	91.00 ± 23.63	140.60 ± 41.96	< 0.001	< 0.001	< 0.001
****P-value	< 0.001	0.16			
**Total cholesterol (mg/dl)**					
Baseline	194.88 ± 38.99	176.24 ± 40.45	0.06		
After intervention	149.00 ± 34.12	180.94 ± 41.55	0.001	< 0.001	< 0.001
****P-value	< 0.001	0.21			
**Low-density lipoprotein- cholesterol (mg/dl)**					
Baseline	121.13 ± 39.67	100.97 ± 41.20	0.05		
After intervention	81.13 ± 32.83	105.60 ± 41.34	0.01	< 0.001	0.01
****P-value	< 0.001	0.71			
**High-density lipoprotein- cholesterol (mg/dl)**					
Baseline	44.72 ± 3.76	47.27 ± 3.01	0.01		
After intervention	50.18 ± 2.12	47.21 ± 3.13	< 0.001	< 0.001	< 0.001
****P-value	< 0.001	0.85			
**Fasting blood sugar (mg/dl)**					
Baseline	100.03 ± 12.98	93.48 ± 11.92	0.06		
After intervention	74.00 ± 4.33	94.03 ± 11.942	< 0.001	< 0.001	< 0.001
****P-value	< 0.001	0.07			
**Insulin (µIU/ml)**					
Baseline	9.57 ± 4.14	11.06 ± 0.07	0.15		
After intervention	6.68 ± 3.21	11.06 ± 4.20	< 0.001	< 0.001	0.01
****P-value	< 0.001	0.98			
**HOMA-IR**					
Baseline	2.38 ± 1.11	2.54 ± 1.03	0.55		
After intervention	1.23 ± 0.63	2.55 ± 1.02	< 0.001	< 0.001	< 0.001
****P-value	< 0.001	0.16			
**HOMA-B**					
Baseline	31.11 ± 14.82	40.13 ± 14.82	0.03		
After intervention	28.92 ± 14.87	39.92 ± 18.64	0.01	0.15	0.49
****P-value	< 0.001	0.09			

− Abbreviations: HOMA-IR: homeostatic model assessment for insulin resistance; HOMA-B: HOMA of β-cell function.

− Values are expressed as means ± SD.

− * Independent sample t-test or Mann-Whitney test were used for comparison of quantitative variables between the two groups.

− **Analysis of covariance (ANCOVA) was used for comparisons between the two groups post-intervention after adjusting for baseline values.

− ***Analysis of covariance (ANCOVA) was used for comparisons between the two groups post-intervention after adjusting for baseline values plus other confounders such as age, weight changes (except for body weight, body mass index, and percent body fat), and physical activity changes.

− ****Paired t-test or Wilcoxon Signed Ranks test were used to compare within group difference from baseline to post-intervention period.

− *P* < 0.05 was considered statistically significant.

## Discussion

This clinical trial has been conducted to determine the effectiveness of a combination of licorice supplementation and a low-calorie diet on obesity and glycemic indices and lipid profiles in overweight/obese women with PCOS. The study showed beneficial effects of this intervention in the improvement of body weight, BMI, FM, FBS, insulin levels, HOMA-IR, and lipid profiles (TC, TG, LDL-C, and HDL-C) compared to a low-calorie diet alone. Also, we did not find any beneficial effects of a low-calorie diet alone against cardiovascular risk factors in overweight/obese women with PCOS, which highlights the importance of complementary therapies in the management of this condition.

In this study, we found that supplementation with licorice extract significantly improved obesity indices compared to the placebo group. However, previous clinical trials and animal research showed inconsistent results following intervention with different types of licorice supplements. In accordance with our results, in a study by Tominaga et al., supplementation with licorice flavonoid oil (LFO) for 12 weeks reduced weight gain in overweight subjects with unhealthy lifestyles [24]. In another study, A 12-week intervention with LFO (300 mg/day) significantly reduced WC and visceral fat in the U.S. population [25]. The results of this study were also confirmed in an animal study that showed adding 1 and 2% LFO to the diet for 8 weeks significantly retarded weight gain and reduced abdominal white adipose tissue in obese mice [26]. In contrast to ours, as reported by Alizadeh et al., licorice supplementation combined with a low-calorie diet did not reduce obesity-related indices such as BMI, WC, and body fat among overweight/obese adults better than a low-calorie diet alone [18]. Also, supplementation with LFO at a dosage of 300 mg/day didn’t reduce anthropometric measurements (body weight, FM, and WC) compared to a placebo after 8 weeks [17]. Moreover, in patients with nonalcoholic fatty liver disease (NAFLD), supplementation with 2 g/day aqueous licorice extract didn’t change BMI compared to placebo after 8 weeks [27]. There may be differences in results between these studies because of differences in intervention duration, control group type, and licorice supplement dose and type.

Several possible mechanisms can explain the effects of licorice supplementation on obesity indices and body composition from a cellular and molecular perspective. Licorice contains glycyrrhizin and glycyrrhetic acid that was found to be involved in energy metabolism and fat distribution by inhibiting the 11β-hydroxysteroid dehydrogenase enzyme at the adipocyte level [28]. Also, licorice affects the gene expression of enzymes related to the fatty acid oxidative pathway in the liver [28, 29] and found to be effective in suppressing food cravings [30].

In this study, the findings of between-group comparisons indicated that licorice supplementation significantly improved FBS, insulin levels, and HOMA-IR compared to placebo after 8 weeks of intervention. Interestingly, our study revealed that licorice supplementation has beneficial effects on IR as a dependent factor associated with PCOS disease severity [8]. There is evidence that hyperinsulinemia, which is associated with compensatory IR and elevated blood glucose, plays a major role in this disease [31]. In women with PCOS, there has been no study examining the effects of licorice supplementation on glycemic indexes and insulin sensitivity; however, a study found that women with PCOS benefit significantly from supplementation with 10 µMg of glabridin, a major ingredient of licorice [19]. In another study by Rostamizadeh et al., supplementation with powder of licorice extract (1000 mg/day) improved serum insulin and IR compared to placebo in patients with NAFLD after 12 weeks [32]. Based on an animal study, the evidence showed that intervention with licorice flavonoid (300 mg/day) reduced FBS and insulin levels in type 2 diabetic rats after 5 weeks [33]. It was also found that 28 days of intervention with galbirdin (40 mg/kg/day) significantly improved FBS and IR in a rat model of diabetic disease [34]. In contrast, other studies found non-significant effects of licorice supplementation in the improvement of glycemic indices and insulin sensitivity [18]. Accordingly, in a study by Alizadeh et al. supplementation with licorice extract in combination with a calorie-restricted diet didn’t decrease FBS, insulin levels, and HOMA-IR compared to a low-calorie diet alone [18]. In another study, supplementation with LFO (1800 mg/day) was found not to be effective in the reduction of FBS and serum insulin levels after 12 weeks [24].

As stated, there are inconsistencies in the findings on licorice supplementation’s effects on glucose homeostasis. This inconsistency might be because of differences in the duration of the studies, type of supplement, disease condition, and type of control groups. It is possible that the reduction in obesity indices following licorice supplementation could be one of the reasons for the improvements in glycemic indices and IR in our study [35]. As stated, obese women with PCOS have higher IR than healthy women with the same BMI, and weight reduction is a priority in the management of PCOS complications [36]. Also, Licorice improves glycemic hemostasis by increasing GLUT4 receptors, AMPK and Akt phosphorylation, and insulin secretion [34]. Moreover, Yoshioka et al. reported that licorice promotes the phosphorylation of mTOR and p70S6K, decreases the phosphorylation of FKHRL1, and reduces atrogin-1 and muscle-specific ring finger protein 1 [37]. Finally, previous research found that 5–40 mg/mL licorice led to dose-dependent inhibition of α-glucosidase [38].

Regarding lipid profiles, the present study showed that licorice supplementation in combination with a low-calorie diet led to significant improvement of TC, TG, HDL-C, and LDL-C compared to a low-calorie diet alone. There has been no study investigating licorice supplementation’s effects on lipid profile in PCOS women, however, a study found that glabridin supplementation didn’t improve lipid profile in PCOS women after 12 months [19]. In contrast to ours, that study had only one arm and intervened with glabridin not licorice in overweight women with PCOS. The effects of licorice supplementation on lipid profiles in other health conditions were inconsistent in some human studies [14, 15, 17, 39, 40]. Accordingly, Mirtaheri et al. studied licorice supplementation in combination with a low-calorie diet that significantly improved TC and LDL-C but not TG and HDL-C [14]. In another study by Fuhrman et al., 0.1 g/d licorice root extract significantly decreased LDL-C and TG levels after one month [15]. Furthermore, supplementation with LFO was found not to be effective in the reduction of lipid profiles in other studies [17]. In an animal study, freeze-dried licorice powder at 100 and 400 mg/day decreased TG and TC levels in a dose-dependent manner [39]. Also, a study demonstrated that ethanolic extract of licorice root decreased TG, TC, VLDL-c, LDL-c, and increased HDL-c levels [40]. Differences in the dietary intake, type of control group, dose and type of licorice supplements, and duration of interventions might result in the contrary results.

The mechanisms by which licorice improves lipid profiles have not yet been clarified, but the reduction in obesity indices following licorice supplementation could be one possible mechanism [41]. Also, the evidence showed that Glabridin, a peroxisome proliferator-activated receptor-α (PPAR-α) agonist, can increase resting metabolic rate [42].

This study had some strengths. It was a double-blind placebo-controlled trial with a high rate of adherence. Also, the analyses were adjusted for some possible confounders. The study also had some limitations. Firstly, the study had a short duration and did not examine the effect of licorice supplementation alone (licorice supplementation without a weight loss diet). Secondly, plasma concentrations of licorice ingredients such as Glabridin and antioxidant enzymes were not evaluated to better clarify underlying mechanisms involved in glucose homeostasis and regulation of lipid profiles by the licorice. Thirdly, 24-hour dietary recall was used for dietary intake assessment which can be attenuated by memorial status and fidelity of the study subjects [43].

## Conclusions

The present study investigated the effects of licorice supplementation in combination with a low-calorie diet in overweight/obese women with PCOS. The findings showed that licorice consumption leads to improvements in obesity indices, glucose homeostasis, and lipid profiles compared to placebo. Due to possible limitations of the study, further research is needed to confirm these findings.

## Data Availability

The data that support the findings of this study are available from the corresponding author upon reasonable request.
